# Automedication in utentes of the municipality of Almada

**DOI:** 10.1080/07853890.2021.1896892

**Published:** 2021-09-28

**Authors:** Maria João Hilário, Sérgio Valério

**Affiliations:** a Escola Superior de Saúde Egas Moniz, Caparica, Portugal; b Lisbon North Universitary Hospital Center, Lisbon, Portugal

## Abstract

**Introduction:**

With the evolution of accessibility to medication in the 1970s and 1980s, self-medication (SM) was generalised, becoming an integral part of health self-care – an independent act of prevention, diagnosis and treatment of the diseases of self without professional counselling [1,2]. In a more global concept, SM may be considered as the act by which the individual, on his own initiative or through the influence of others, decides to use a drug for relief or treatment of self-reported grievances [3]. This concept essentially encompasses two forms: responsible SM where the individual should be informed about the non-prescription medicines (MNSRM) through the leaflets and health technicians, so that they use it efficiently and safely. Non-responsible SM is one in which there is no information about MNSRM and where self-diagnosis occurs [4]. The objectives of the study are: verify the percentage of SM and characterise it in the adult users of the municipality of Almada, verify if there is a relation between the practice of SM with the sociodemographic variables – age, sex and literacy.

**Materials and methods:**

Type of study: descriptive-correlational. The sample was non-probabilistic, accidental. 321 inhabitants of the municipality of Almada were inquired, aged over 16 years, when leaving pharmacies between May and June 2018. A 90% confidence interval and 1.88 standard deviations were used. The information was collected through a survey with 14 questions asked directly to subjects, which was validated with 20 pre-tests. All respondants gave their consent when answering to the survey.

**Results:**

Percentage of SM: 50.16%; Mean of participants was 55 years old and standard deviation of 17.2. 113 cases for the feminine gender, 48 cases for male gender; 35 cases for age (57–66) as the most representative class; Spearman corr. where: between gender and SM, *r*_s_= 0.101, *p* < .01, positive and significant; between SM and literacy *r*_s_=–0.350, *p* < .001, negative and significant; between SM and age *r*_s_= 0.422, *p* < .001, positive and significant.

**Discussion and conclusions:**

It is verified that the percentage of self-medication is about half of the respondents, being superior in the female sex and more represented by the age group of 57–66 years. There is direct and significant relation between SM and gender and SM and age. On the other hand there is an inverse and significant relation between SM and literacy.
